# Natural killer T cell activation increases iNOS^+^CD206^-^ M1 macrophage and controls the growth of solid tumor

**DOI:** 10.1186/s40425-019-0697-7

**Published:** 2019-08-06

**Authors:** Sourav Paul, Sushanta Chhatar, Amrita Mishra, Girdhari Lal

**Affiliations:** National Centre for Cell Science, NCCS Complex, Pune University Campus, Ganeshkhind, Pune, MH-411007 India

**Keywords:** Tumor microenvironment, Macrophage polarization, Th1, Natural killer T cells, CD1d, Melanoma

## Abstract

**Background:**

NKT cells play an important role in anti-tumor immunity. Alpha-galactosylceramide (α-GalCer), a synthetic glycolipid is presented to natural killer T (NKT) cells by most antigen-presenting cells through CD1d molecules leading to activation of NKT cells. However, the precise mechanisms of how α-GalCer-activated NKT regulate the polarization of the macrophages and effector T cells in the solid tumor are not studied adequately.

**Methods:**

We induced solid tumor in C57BL/6 mice by subcutaneous injection of B16F10 cell line (1 X 10^6^ cells) and monitored the tumor growth. Animals were given an intraperitoneal injection of α-GalCer (2 μg/injection) in 200 μl PBS on day + 1, + 5, + 10, + 15, and + 20 (with respect to tumor cell injection). Immune cells were characterized using flow cytometry and immunofluorescence staining. NK cells, Gr1^+^ cells, and F4/80^+^ macrophages in the mice were depleted by intravenous injection of cell-specific antibodies. Statistical analysis was performed using Student’s *t*-test or one-way ANOVA.

**Results:**

Our results showed that intratumoral NKT cells have a lower frequency of CD69, CD25, CD122, and IFN-γR expression; produced less inflammatory cytokines such as IFN-γ, TNF-α, and GM-CSF; higher frequency CD62L^+^ NKT cells; and also showed reduced proliferation as compared to the splenic NKT cells. Mice treated with α-GalCer showed a significantly increased frequency of IFN-γ-producing NKT cells, CD8^+^ T cells, and effector Th1 cells. Depletion of NK cells in α-GalCer-treated mice showed a lower frequency of IFN-γ-producing CD4^+^ and CD8^+^ T cells in the tumor and prevented the α-GalCer-induced tumor growth. NKT cell activation with α-GalCer treatment significantly increased the iNOS^+^CD206^−^ M1-macrophages and reduced the iNOS^−^CD206^+^ M2-macrophages in the spleen and tumor, and depletion of F4/80^+^ macrophages prevented the α-GalCer-induced reduction in the tumor growth.

**Conclusions:**

We showed that activation of NKT cell with α-GalCer modulates the frequency of M1-macrophages and effector Th1 cells in the secondary lymphoid tissues and tumor microenvironment and inhibit tumor growth. The finding suggests that activation of NKT cells with α-GalCer may provide an effective anti-cancer outcome.

**Electronic supplementary material:**

The online version of this article (10.1186/s40425-019-0697-7) contains supplementary material, which is available to authorized users.

## Introduction

Natural killer T (NKT) cells show the characteristics of innate as well as adaptive immune cells. These cells express T cell receptor (TCR) and respond to self- or non-self-lipid antigens loaded on CD1d molecules. NKT cell activation leads to rapid production of inflammatory cytokines and modulates the function of several effectors and regulatory immune cells both in mice and humans [1, 2]. According to the nature of the activating ligand, NKT cells are classified into two groups; type-I and type-II NKT cells. The type-I NKT cells (also known as invariant NKT cells or iNKT cells) express semi-invariant Vα14-Jα18 TCR chain in mice and Vα24-Jα18 chains in humans, and recognize self- or microbial-lipids presented by CD1d molecules [1, 3]. The type-I NKT cells constitute about 0.2–2% of lymphocytes in the murine bone marrow, thymus, blood, and spleen, and about 0.05–1% in the human peripheral blood [4]. Type-I NKT cells are reported to produce regulatory cytokines (e.g., IL-4 and IL-10) or pro-inflammatory cytokines (e.g., IL-2, IL-17, TNF-α, and IFN-γ) [4]. The type-II NKT cells are also CD1d-restricted but do not express the invariant Vα14-Jα18 TCR chain. These NKT cells show diverse TCRα and β chain and recognize sulfatide or lysophosphatidylcholine (LPC) antigens and inhibit the pro-inflammatory function of type I NKT cells [5–7]. PBMCs from multiple myeloma and breast cancer patients show a decreased frequency of NKT cells as compared to the healthy individual [8, 9]. Whereas, colorectal tumor patients show a high NKT cell infiltration and serve as a useful prognostic marker for overall survival of colorectal cancer patients [10]. The patients with head and neck squamous cell carcinoma show the reduced number of circulating NKT cells and is associated with a lower survival rate of the patients [11].

α-galactosylceramide (α-GalCer, also known as KRN7000) is presented by CD1d molecule and act as a potent activator of type-I NKT cells but not type-II NKT cells [7]. α-GalCer induces IFN-γ production in the NKT cells [12–15] and treatment with α-GalCer control the liver metastasis in mice [16]. Adoptive transfer of α-GalCer-pulsed dendritic cells (DC) inhibits the liver metastasis in an NKT cell-dependent manner [17]. Furthermore, adoptive transfer of in vitro-expanded NKT cells with α-GalCer-pulsed DC offers protection from the lung metastases [18]. IFN-γ production by NK and NKT cells play a critical role in the anti-metastatic effect of α-GalCer in the liver metastasis [19]. Comparatively, liver NKT cells can also confer better protection against MCA-induced fibrosarcoma than splenic- or thymus-derived NKT cells [20].

Macrophages play an important role in the tumor microenvironment and modulate the function of other immune cells. Macrophages are classified into two major groups- classical M1 macrophages and alternative M2 macrophages [21]. M1 macrophages mostly produce pro-inflammatory molecules such as TNF-α, IL-6, IL-12, IL-23, and inducible nitric oxide synthase (iNOS). These macrophages can also present tumor-specific antigens to T cells and help in the anti-tumor immunity. In contrast, M2 macrophages secrete Th2-type cytokines such as IL-4, IL-10, IL-13, and TGF-β, and show a pro-tumorigenic phenotype [21]. Majority of tumor-associated macrophages (TAM) are of M2 phenotype, and have poor antigen presentation capacity and can suppress the T cell response [22].

NKT cells infiltration in the tumor microenvironment are very well documented [1, 23–25], however, what are the phenotype of these intratumoral NKT cells, and how they interact with other immune cells in the tumor microenvironment to control tumor growth, is not very clear. Most of the studies on NKT cells have used metastatic than a solid tumor model. However, the mechanisms underlying the effect of α-GalCer on NKT cell phenotype and function of CD4^+^ T cells, CD8^+^ T cells, and macrophages are not entirely understood. In the present study, we showed that B16F10 cell-induced tumor have higher infiltration of CD62L^hi^ NKT cells. These intratumoral NKT cells produced lower IFN-γ, TNF-α, and GM-CSF as compared to splenic NKT cells. The treatment with α-GalCer increase the frequency of IFN-γ-producing CD1d-tetramer^+^-NKT cells, effector CD8^+^ T cells, and Th1 cells in the tumor and spleen. Furthermore, treatment with α-GalCer increased the frequency of M1 macrophages in the tumor leading to reduced tumor growth.

## Materials and methods

### Antibodies and reagents

Alexa Fluor 488 NK1.1 (PK136), Biotin-CD4 (GK1.5), Alexa Fluor 488-CD4 (GK1.5), APC-eFluor 780-CD4 (GK1.5), FITC-CD3ε (17A2), Alexa Fluor 647-CD3ε (17A2), Alexa Fluor 488-CD206, Brilliant Violet 421-CD3ε (17A2), FITC-F4/80, Alexa Fluor 488-CD3ε (145-2C11), Alexa Fluor 647 CD49b (DX5), Pacific Blue-CD49b (DX5), PE-NK1.1 (PK136), Brilliant Violet 421 NK1.1 (PK136), PE/Cy5-CD4 (GK1.5), APC-TCRγδ (GL3), Biotin-IFN-γRα (2E2), Brilliant Violet 421-CD25 (PC61), PE-CD25 (PC61), Biotin-CD122 (5H4), PE/Cy7-IFN-γ (XMG1.2), PE-GM-CSF (MP1-22E9), Pacific Blue-TNF-α (MP6-XT22), PercCP/Cy5.5-CD69, Biotin-BrdU (Bu20a), FITC-Ki67 (16A8), Alexa Fluor 647-streptavidin, APC-Cy7-Streptavidin, PE-Cy7-Streptavidin, Brilliant violet 421-CD62L (MEL-14) and rabbit polyclonal anti-Asialo-GM1 (aGM) antibodies were purchased from Biolegend (San Diego, CA). APC-RORγt (AFKJS-9), APC-T-bet (4B10) and PE/Cy7-T-bet (4B10) were procured from eBioscience (San Diego, CA). Anti-NK1.1 (PK136), anti-F4/80 (Cl:A3–1), anti-Gr1 (RB6-8C5) and isotype control antibodies for in vivo use were procured from BioXcell (West Lebanon, NH). Alexa Fluor 647-labeled CD1d tetramer (mCD1d, PBS-57) and unloaded control tetramer were received from NIH Tetramer Core Facility (Atlanta, GA). 5-Bromo-2′-deoxyuridine (BrdU) was purchased from Sigma-Aldrich.

### Mice

Six to eight-weeks-old C57BL/6 male mice were used. These mice were procured from Jackson Laboratory (Bar Harbor, Maine), and bred in the National Centre for Cell Science (NCCS) experimental animal facility.

### Induction of tumor in mice

Mouse melanoma B16F10 cell line was received from the national cell repository of National Centre for Cell Science, Pune, India, and maintained in complete high-glucose DMEM culture medium [DMEM with 10% FBS (Gibco), NaHCO_3_ (1.5 g/liter), penicillin (50 units/ml), streptomycin (50 μg/ml) and sodium pyruvate (1 mM)] at 37 °C in a humidified 5% CO_2_ incubator. C57BL/6 mice were given a subcutaneous injection of B16F10 cells (1 X 10^6^ cells). The tumor area was measured using a caliper every alternate day. Tumor area was calculated as A = L x W, where L = length of tumor (in mm), W = width of tumor (in mm), A = area (in mm^2^). To test the effect of α-GalCer on the tumor growth, intraperitoneal injection of 2 μg of α-galactosylceramide (α-GalCer, also known as KRN7000, Cayman Chemical Company, Ann Arbor, MA) in 200 μl PBS was given on day + 1, + 5, + 10, + 15 and + 20 (with respect to tumor cell injection).

### Isolation and staining of cells

Single cell suspension was prepared as described earlier [26]. Tumors were excised, manually disrupted into small pieces using fine forceps, re-suspended in 1X Hanks balanced-salt solution (HBSS) containing collagenase type I (0.1 mg/ml, Gibco) and collagenase type IV (0.1 mg/ml, Sigma), hyaluronidase (0.06 mg/ml, Sigma), DNase I (0.02 mg/ml, Sigma) and soybean trypsin inhibitor (0.1 mg/ml, Sigma). The cell suspension was incubated at 37 °C in a shaking water bath for 30–90 min. Then, the cell suspension was passed through a 70 μm pore-containing cell strainers (BD Biosciences, San Jose, CA). RBC was removed using ACK lysis buffer and washed with RPMI medium. The single cell suspension was used for flow cytometry sorting (FACS Aria III, BD Bioscience) or phenotypic analyses (FACS Canto II, BD Bioscience).

The single cell suspension of spleen and lymph nodes were prepared by mechanical disruption and passing the cell suspensions through a 70 μm pore-containing cell strainers. RBCs were removed by ACK lysis buffer, washed with RPMI 1640 medium, stained and re-suspended in RPMI 1640 medium and used for flow cytometry analysis.

### Intracellular cytokine staining

Intracellular cytokine staining was performed as described earlier [26]. Briefly, cells were stimulated with 81 nM PMA, 1.34 μM ionomycin, 10.6 μM brefeldin and 2 μM monensin in complete RPMI medium at 37 °C in 5% CO_2_ incubator for 6 h. Cells were washed and surface-stained using saturating concentration of specific antibodies on ice for 30 min; washed and incubated with appropriate secondary reagents (1:500 dilution) on ice for 30 min. Intracellular cytokines and transcription factors staining were performed using Foxp3 Fixation/permeabilization kit (Biolegend, San Diego, CA) according to the manufacturer’s instructions.

### In vivo depletion of NK1.1^+^ cells and F4/80^+^ macrophages

NK1.1^+^ cells were depleted by intravenous (*i.v.*) injection of anti-mouse NK1.1 monoclonal antibody (clone PK136; 100 μg/injection/mouse; Bioxcell, West Lebanon, NH) or anti-asialo GM1 polyclonal antibody (anti-aGM1; 100 μg/injection/mouse; eBioscience) on day − 3, + 1, + 5, + 10, + 15 and + 20 with respect to tumor cell injection. Mice were sacrificed either on day 13 or 23, and NK1.1^+^ cell depletion was monitored using flow cytometry. Depletion of NK1.1^+^ cells in the spleen was > 96% **(**Additional file 1: Figure S1**)**. The depletion of NK cells using anti-NK1.1 antibody did not affect the frequency of Foxp3^+^ Treg, γδ T cells, F4/80^+^ macrophages, myeloid-derived dendritic cells, and DCs (61) **(**Additional file 1: Figure S1). Macrophages were depleted by intravenous (*i.v.*) injection of anti-F4/80 monoclonal antibody (clone Cl:A3–1; 100 μg/injection/mouse; Bioxcell, West Lebanon, NH) or monocytes with anti-Gr1 monoclonal antibody (clone RB6-8C5; 100 μg/injection/mouse; Bioxcell, West Lebanon, NH) on day − 3, + 1, + 5, + 15 and + 20 with respect to tumor cell injection.

### Immunofluorescence staining for microscopy

Spleen and tumor tissues were harvested, cut into small pieces, embedded in OCT freezing medium (Sakura Finetek, Torrance, CA), and stored in − 80 °C until further use. Tissue sections (8 μm thick) were fixed in chilled acetone for 5 min, air dried, washed with cold PBS, and blocked with 10% normal horse serum (Jackson ImmunoResearch, West Grove, PA) at room temperature (RT) for 30 min. Then washed with PBS and incubated with indicated fluorochrome-conjugated primary Abs (1:200 dilution for FITC anti-mouse F4/80, 1:400 dilution for rabbit monoclonal Ab to iNOS (Abcam) and 1:100 dilution for Alexa Fluor 488 anti-mouse CD206, and Alexa Fluor 647 anti-mouse F4/80) on ice for 45 min. Followed by three-time washing with cold PBS, and incubated with secondary antibodies (1:1000 dilution for donkey Dylight 549 anti-rabbit antibody) for 30 min; then washed three-times with PBS, fixed with 1% paraformaldehyde, and mounted in aqueous mounting medium containing DAPI (Electron Microscopy Sciences, Hatfield, PA). Immunofluorescence images were captured using the Leica DMI 6000 fluorescent microscope (Leica Microsystems, Germany), and data were analyzed using Leica AF6000 software.

### Statistical analysis

Statistical analysis of data was performed by Unpaired two-tailed Student’s *t-*test and one-way analysis of variance (ANOVA) where appropriate was used to compare two independent groups. All statistical analyses were performed using GraphPad Prism 7 and Prism 8 software (GraphPad Software, San Diego, CA). (*p < 0.05; **p < 0.01; ***p < 0.001; and ****p < 0.0001; ns, not significant). *p* < 0.05 was considered significant.

## Results

### Activation and proliferation status of intratumoral NKT cells

**Fig. 1:**
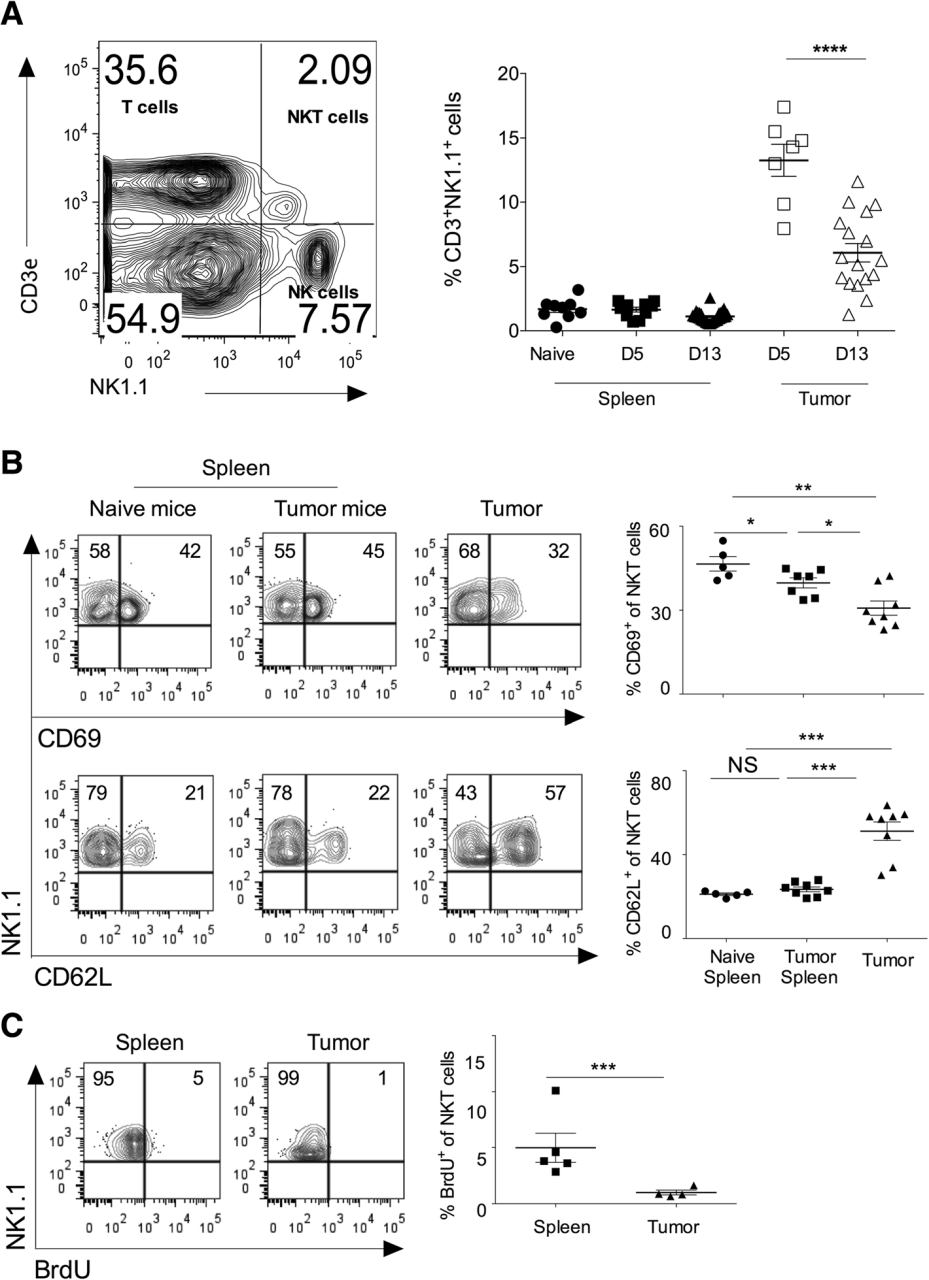
Intratumoral NKT cells show increased CD62L expression, and low activation marker and proliferation. B16F10 cells (1 X 10^6^ cells/mouse) were *s.c.* injected in the naïve C57BL6 mice. **a** On day 5 and 13 of B16F10 injection, CD3^+^NK1.1^+^ cells were analyzed using flow cytometry. A representative dot plot showing the NKT cell population is shown (left panel). Cells shown in the dot plots are gated on the lymphocytic gate (based on FSC-A vs. SSC-A scatter) followed by singlet populations (FSC-A vs. FSC-W scatter). Numbers in the dot plot indicate the percentage of cells. The mean percentage of NKT cells in the spleen and tumors are plotted (right panel). *n* = 8–10 mice/group for day 5; and *n* = 17 mice/group for day 13. **b** At day 13, NKT cells were analyzed. The dot plots showing CD69 and CD62L expression after gating on NKT cells (left). The bar represents mean, and each dot represents individual mouse (right). *n* = 5–8 mice/group. **c** B16F10 cells (1X10^6^ cells/mouse) were s.c. injected in the naïve C57BL6 mice, and also intraperitoneally given BrdU (150 μg/mouse) twice a day for three constitutive days. At day 15, immune cells were stained with anti-BrdU mAb and analyzed after gating on NKT cells (left). The error bar represents s.e.m., and each dot represents data from an individual mouse (right). *n* = 4–5 mice/group. Student’s *t*-test (**a**, **b**, **c**). In all panels, *p < 0.05; **p < 0.01; ***p < 0.001; ****p < 0.0001; NS, not significant

Subcutaneous injection of B16F10 melanoma cells in syngeneic C57BL/6 mice showed the progression of tumor growth, and tumor showed infiltration of mononuclear cells, including CD3^+^NK1.1^+^ (NKT) cells (Fig. 1a). On day 5 of B16F10 cell injection, CD3ε^+^NK1.1^+^NKT cells comprise about 14% of the total infiltrating lymphocytes (Fig. 1a). On day 13, intratumoral NKT cells frequency was significantly reduced (Fig. 1a). Cell adhesion molecules regulate the recruitment of NKT cells into the tumor microenvironment. CD62L^+^ NKT cells show prolonged persistence within tumors and are also reported to have anti-tumor activity [27]. Consistent with these reports, we observed that intratumoral NKT cells had a significantly higher frequency of CD62L^+^NKT cells as compared to splenic NKT cells, indicating that CD62L might help in the accumulation of NKT cells in the tumor microenvironment (Fig. 1b). CD69 is an early activation marker, and we tested the activation status of intratumoral NKT cells. Analysis of intratumoral NKT cells revealed that these cells had significantly reduced expression of CD69 as compared to splenic subsets indicating that intratumoral NKT cells have lower activation phenotype (Fig. 1b). Since our results showed infiltration of NKT cells in the tumor, we examined whether this was due to the local proliferation of intratumoral NKT cells or its recruitment from the peripheral tissues. To this end, C57BL/6 mice were given BrdU twice a day for three days intraperitoneally, and incorporation of BrdU in the NKT cells was monitored using flow cytometry. Our data showed that intratumoral NKT cells had significantly reduced incorporation of BrdU as compared to the splenic subset (Fig. 1c). Together**,** these results suggest that intratumoral NKT cells have increased cell adhesion molecules, reduced activation, and exhibit lower proliferation as compared to splenic NKT cells.

### Cytokines and cytokine receptors expression on the intratumoral NKT cells

**Fig. 2:**
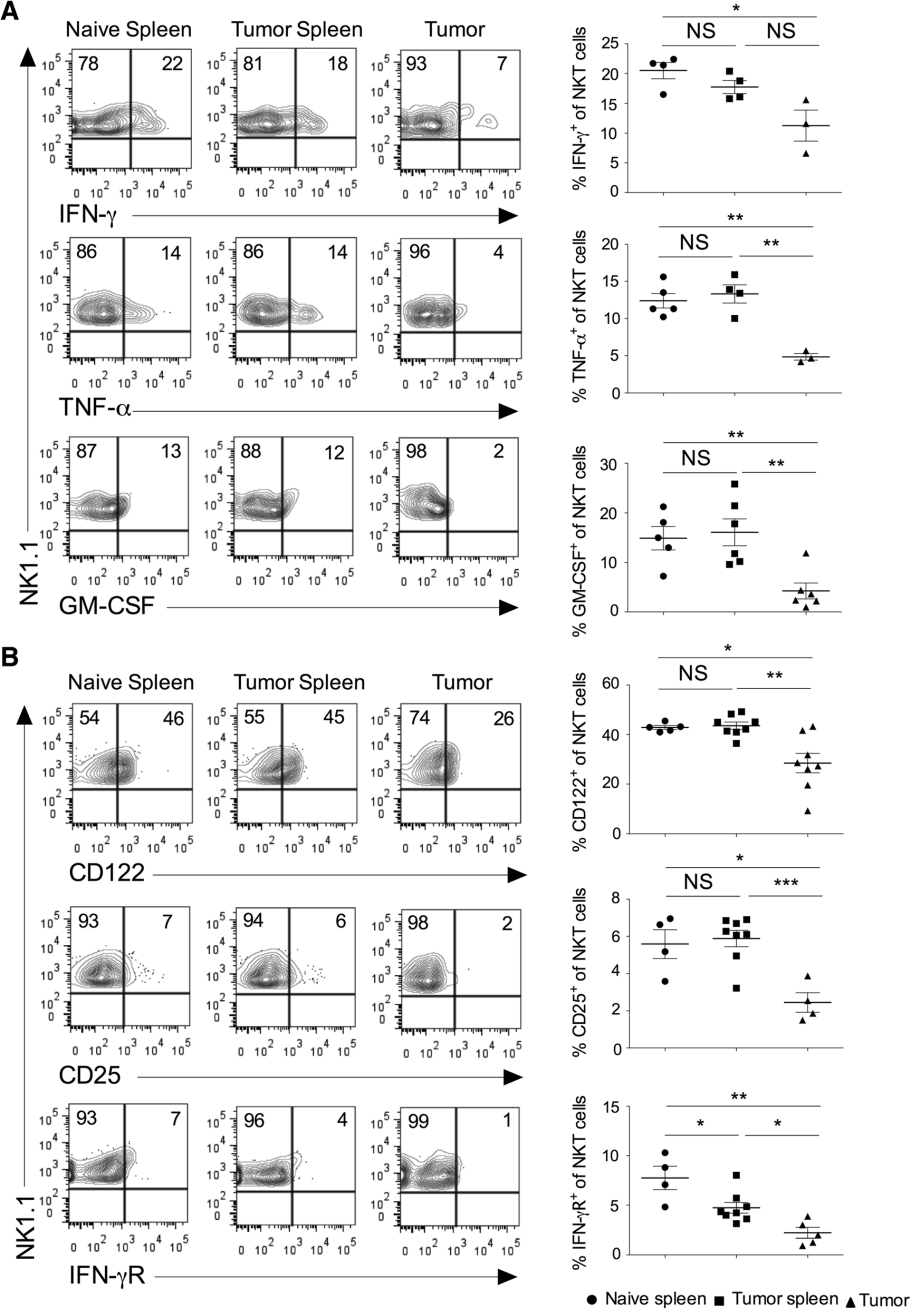
Intratumoral NKT cells show altered expression of cytokines and cytokine receptors*.* Naïve C57BL6 mice were given s.c. injection of B16F10 cells (1 X 10^6^ cells/mouse). **a** At day 13, spleen and tumors were harvested. The single cell suspension was stimulated with PMA/ionomycin, and intracellular cytokines expression was analyzed after gating on NKT cells. The representative contour plots are shown (left panel), and data from all the mice are shown (right panel). *n* = 3–6 mice/group. **b** On day 13, the surface expression of CD25 (IL-2Rα), CD122 (IL-2Rβ) and IFN-γR on NKT cells were analyzed (left). *n* = 4–8 mice/group. The bar represents s.e.m., and each dot represents data from an individual mouse (**a**, **b**). Student’s *t*-test (**a**, **b**). In all panels, *p < 0.05; **p < 0.01; *** *p* < 0.001; NS, not significant

Activated NKT cells are known to produce several cytokines, modulate the function of other immune cells, and affect the tumor growth and metastasis [28–30]. We have analyzed the expression of cytokines in the NKT cells in the spleen and tumor. Our results showed that intratumoral NKT cells secreted significantly lower IFN-γ, TNF-α, and GM-CSF as compared to splenic subsets (Fig. 2a). The expression of IL-4 in the NKT cells did not change between tumor and spleen (data not shown). NKT cells express various cytokine receptors and rapidly respond to the specific cytokine stimulation [1, 31]. IL-15 has been shown to regulate the maturation and survival of NKT cells [32]. Similarly, cytokines such as IL-2, IL-12, and IL-15 induce proliferation and cytotoxic function of NKT cells [33]. IL-12 and IL-18 stimulation in the absence of TCR engagement enhances the production of IFN-γ in NKT cells [34]. Our results showed that intratumoral NKT cells had reduced expression of CD122 (IL-2Rβ), CD25 (IL-2Rα), and IFN-γR as compared to the splenic subsets (Fig. 2b). Collectively, these results suggest that intratumoral NKT cells have decreased inflammatory cytokines secretion and lower expression of cytokine receptors indicating its poor anti-tumor activity.

### Effect of α-GalCer on various cytokines secretion and tumor growth

**Fig. 3:**
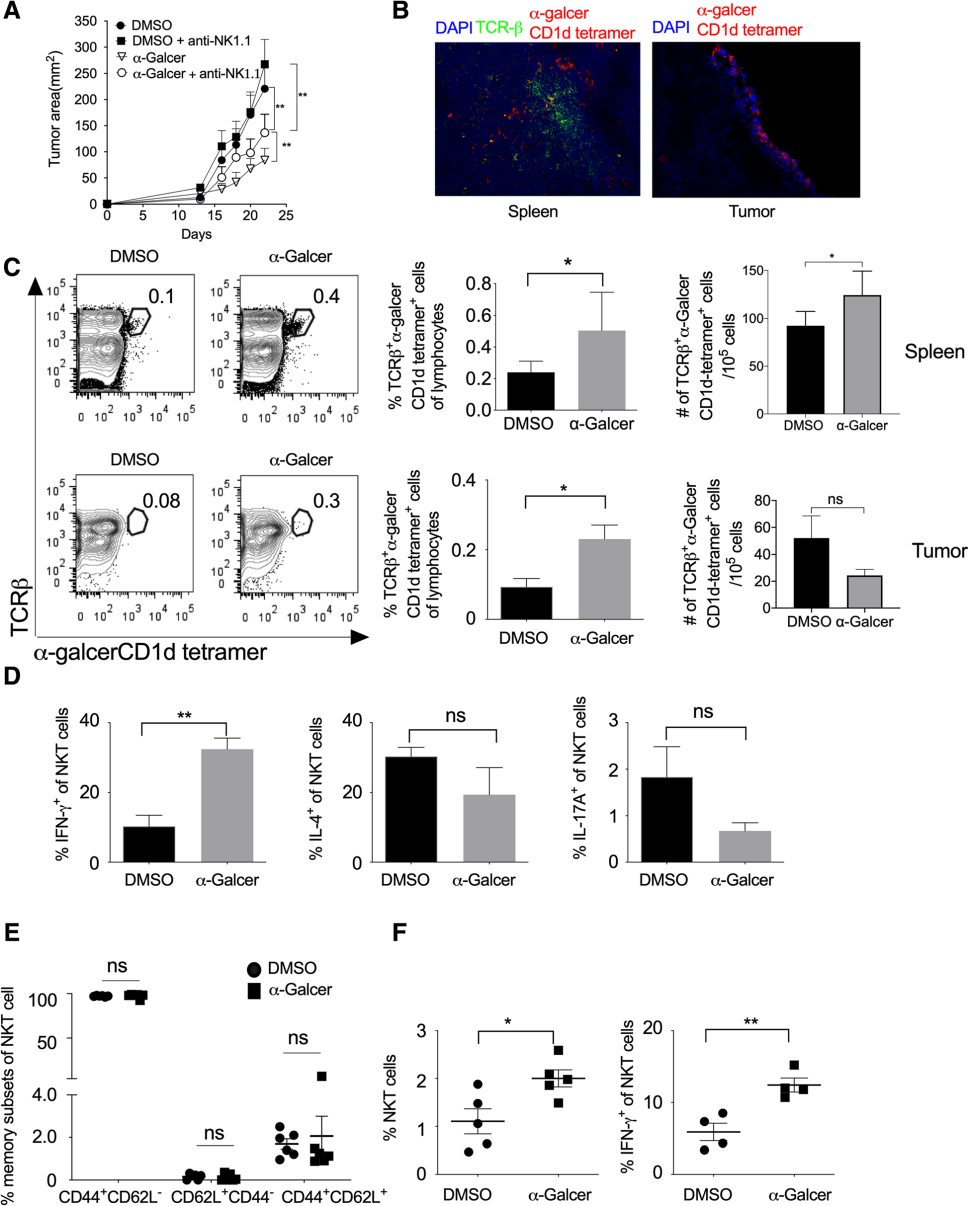
α-GalCer increases the frequency of NKT cells, IFN-γ secretion, and inhibits tumor growth. Naïve C57BL6 mice were given s.c. injection of B16F10 cells (1 X 10^6^ cells/mouse), and animals were also given *i.v.* injection of NK1.1 mAb (PK136; 100 μg/mouse/injection) on day − 3, + 1, + 5, + 10 and + 15 (day with respect to tumor cell injection). α-GalCer (2 μg/mouse/i.p injection) was given on day + 1, + 5, + 10, + 15 and + 20. **a** The tumor area was calculated and plotted. *n* = 6 mice/group. The data shown are representative of two independent experiments. **b** On day 13, spleen and tumor tissues from α-GalCer-treated mice were stained with TCR-β, α-GalCer-loaded CD1d-tetramer and nuclear stain DAPI. Images were acquired using a fluorescent microscope, and the representative images of spleen and tumor from α-GalCer treated mice are shown (magnification 200X). **c** On day 13, NKT cells in the spleen and tumor were analyzed using flow cytometry. Representative contour plots are shown (left panel) and mean percentages of NKT cells are plotted (middle panel). The absolute number of cells were calculated and plotted (right panel) *n* = 5 mice/group. **d** On day 13, IFN-γ, IL-4 and IL-17A expression in the NKT cells in the spleen were analyzed and plotted. *n* = 5–6 mice/group. **e** On day 13, based on CD62L and CD44 expression, memory NKT cell subsets were analyzed and plotted. *n* = 5–6 mice/group. **f** On day 23, the mean percentages of NKT cells and IFN-γ^+^-producing NKT cells in the spleen were analyzed. The bar represents mean, and each dot represents an individual mouse. *n* = 4–5 mice/group. (**b**-**e**). One-way ANOVA (**a**), Student’s *t*-test (**c**-**e**), *p < 0.05; ** *p* < 0.01; ns, not significant

α-GalCer is known to stimulate the type-I NKT cells, which in turn activates and induce the proliferation of other leukocytes [19, 35, 36]. To study the role of NKT cells in the regulation of tumor growth, we subcutaneously (s.c.) injected B16F10 cells in C57BL/6 mice and also administered *i.p.* injection of α-GalCer and monitored tumor growth. Our results showed that α-GalCer treatment significantly reduced B16F10 melanoma tumor size (Fig. 3a and Additional file 1: Figure S2). NKT cells play a very crucial role in controlling tumor growth [26]. To test the effect of NK cells in the α-GalCer-treated mice on tumor growth, B16F10 cells were subcutaneously injected in C57BL/6 mice and treated with α-GalCer. In these mice, NK cells were depleted by intravenous injection of anti-NK1.1 mAb (PK136) and monitored the tumor growth. Although NK cell depletion itself promote the tumor growth in mice [26], our results showed that depletion of NK cells prevented the α-GalCer-induced inhibition of tumor growth (Fig. 3a and Additional file 1: Figure S2**)** suggesting that α-GalCer require NK1.1^+^ cells for its anti-tumor activity. Furthermore, the immunohistological analysis of spleen and tumor tissues showed the presence of α-GalCer-CD1d tetramer^+^ NKT cells (Fig. 3b). On day 13, we found that α-GalCer treatment increased the frequency of α-GalCer-CD1d tetramer^+^ NKT cells in both spleen and tumor, and also had significantly increased in the number of α-GalCer-CD1d tetramer^+^ NKT cells in the spleen (Fig. 3c). Anti-NK1.1 antibody (clone PK136) is known to deplete both NK and NKT cells. To specifically investigate the role of NKT cells on α-GalCer-mediated inhibition of tumor growth in mice, we specifically depleted NK cells using anti-asialo GM1 antibody. This antibody known to depletes only NK cells but not NKT cells. Our results showed that anti-asialo GM1 antibody treatment reduced the α-GalCer-induced reduction of tumor growth (Additional file 1: Figure S3A), however, the anti-asialo GM1 mAb treatment did not affect the frequency of IFN-γ-producing NKT cells in the spleen (Additional file 1: Figure S3B). These results suggest that although α-GalCer activates only NKT cells, α-GalCer-induced inhibition of tumor growth require NK cells. Furthermore, α-GalCer treatment significantly increased IFN-γ production and slightly lowered the expression of IL-4 and IL-17 in the splenic NKT cells (Fig. 3d).

The cord blood NKT cells are known to express CD45RO along with CD62L and CCR7 molecules and display mostly central-memory phenotype [37–39]. Our results showed that α-GalCer treatment did not alter the effector memory subsets of NKT cells as compared to vehicle-treated mice (Fig. 3e). Furthermore, on day 23 of B16F10 cell injection, α-GalCer treatment increased the frequency of total NKT cells as well as IFN-γ-producing NKT cells (Fig. 3f) whereas the frequency of IL-4 or IL-17A-producing NKT cells were not affected in the tumor (data not shown). Together, these results suggest that α-GalCer promotes the frequency of IFN-γ-producing NKT cells in the spleen and tumor and help in controlling the tumor growth.

### Effect of α-GalCer on IFN-γ-producing effector CD8^+^ T cells and Th1 cells

**Fig. 4:**
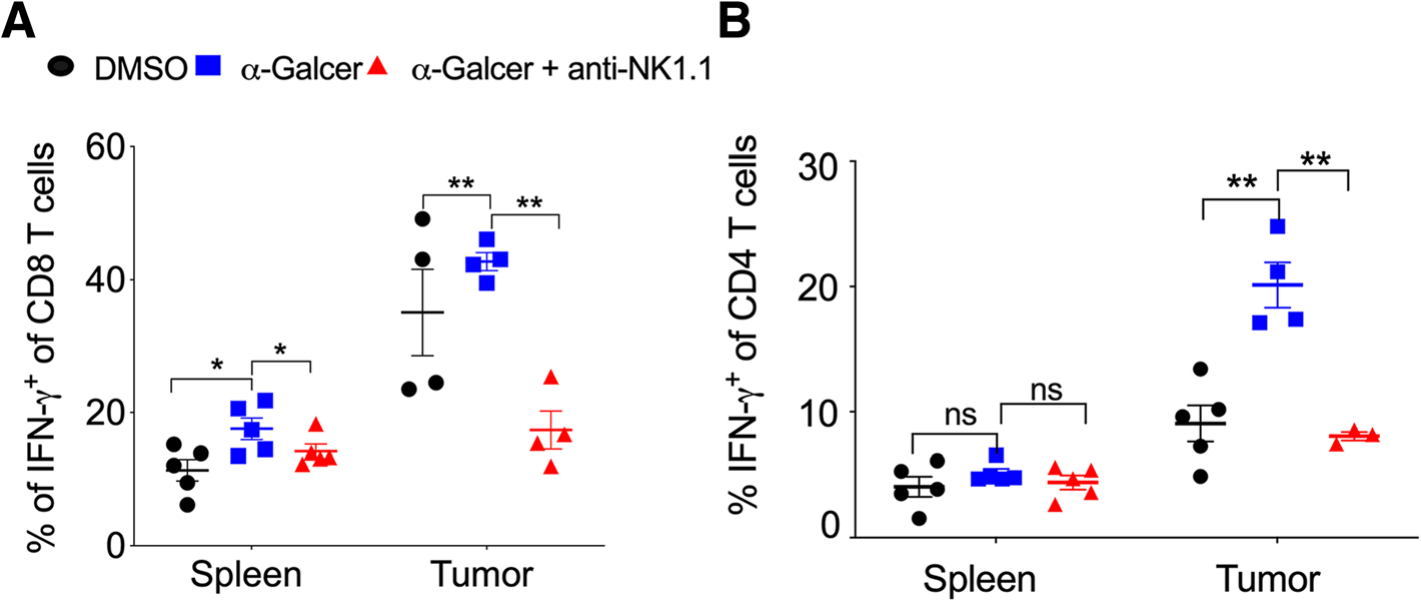
α-GalCer-treatment increase the frequency of IFN-γ-producing CD8^+^ T cells and Th1 cells. Naïve C57BL6 mice were given s.c. injection of B16F10 cells (1 X 10^6^ cells/mouse) and also injected anti-NK1.1 mAb and treated with α-GalCer as in Fig. 3a. **a** At day 13, IFN-γ^+^ production in the splenic and intratumoral CD8^+^ T cells was analyzed after gating on lymphocytic gate followed by singlet populations. *n* = 4–5 mice/group. **b** At day 13, CD4^+^IFN-γ^+^ T cells were analyzed after gating on CD4^+^ cells. *n* = 4–5 mice/group. The bar represents s.e.m., and each dot represents data from an individual mouse. Student’s *t*-test. ** *p* < 0.01, ns, not significant

The effector CD8^+^ T cells and Th1 cells play a very crucial role in controlling tumor growth [40]. We investigated whether α-GalCer had any effect on the frequency of effector CD8^+^ T cells and Th1 cells. C57BL/6 mice were given s.c. injection of B16F10 cells and treated with α-GalCer and monitored the frequency of IFN-γ-producing CD8^+^ as well as CD4^+^ T cells. Our results showed that the frequency of total CD8^+^ and CD4^+^ T cells were unaltered (data not shown). However, in the spleen and tumor, the percentage of IFN-γ-producing CD8^+^ T cells was significantly higher in α-GalCer-treated mice as compared to control mice (Fig. 4a). α-GalCer-induced IFN-γ-production in CD8 T cells in the spleen and tumor was reduced with depletion NK cells (Fig. 4a). α-GalCer treatment did not alter IFN-γ-producing CD4 T cells (Th1) cells in the spleen (Fig. 4b). Interestingly, α-GalCer treatment showed increased intratumoral Th1 cells (Fig. 4b), and it was reduced with anti-NK1.1 mAb treatment (Fig. 4b**)**. There was no change in the percentage of CD4^+^, CD8^+^, and γδ T cell in the spleen and tumor of DMSO control, α-GalCer or α-GalCer plus anti-NK1.1 mAb-treated tumor-bearing mice (data not shown). These results suggest that α-GalCer increase the IFN-γ-producing CD8^+^ T cells in the spleen and tumor and promote the intratumoral Th1 cells.

### Effect of α-GalCer on the intratumoral frequency of M1-macrophage and tumor growth

**Fig. 5:**
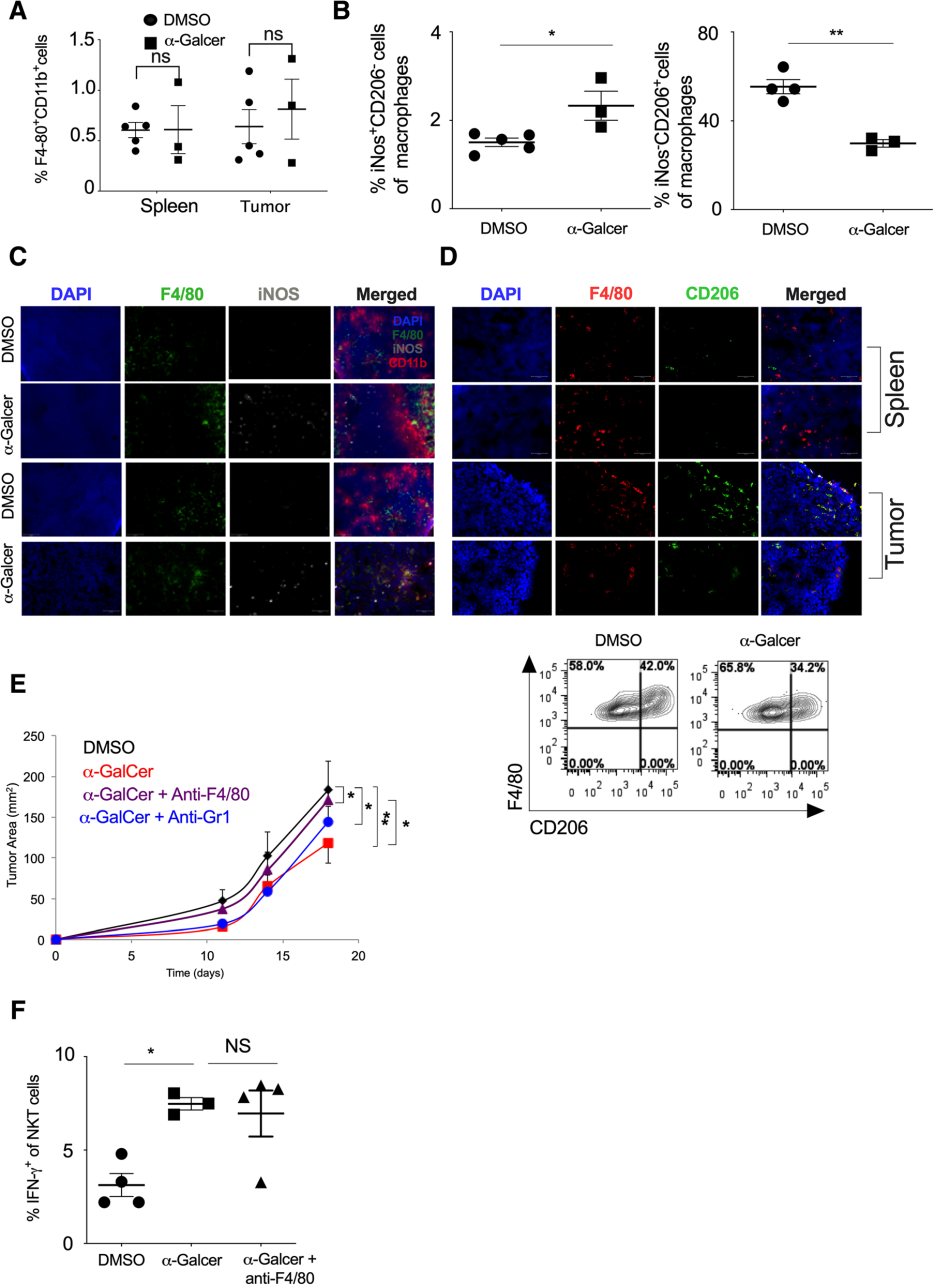
a-GalCer-treated mice show a higher frequency of M1 macrophages and low tumor growth. Naïve C57BL6 mice were given s.c. injection of B16F10 cells (1 X 10^6^ cells/mouse) and animals were treated with α-GalCer injection (2 μg/mouse/i.p injection) on the day + 1, + 5, + 10, and + 15 (day with respect to tumor cell injection). **a** At day 20, the percentage of F4/80^+^CD11b^+^ cells in the spleen and tumor were analyzed. *n* = 3–5 mice/group. **b** At day 20, the percentage of iNOS^+^ cells (M1 macrophage; left panel) and CD206^+^ cells (M2 macrophage; right panel) in the spleen were analyzed after gating F4/80^+^CD11b^+^ cells. *n* = 3–5 mouse/group. **c** iNOS^+^F4/80^+^ cells (M1 macrophage) in the spleen and tumor were analyzed by immunofluorescence microscopy and the representative images are shown. Original magnification 400x. **d** CD206^+^F4/80^+^ cells (M2 macrophage) in the spleen and tumor of DMSO and α-GalCer-treated mice were analyzed by immunofluorescence staining (upper panel). Original magnification 400x. Representative contour plots of CD206^+^F4/80^+^ cells (M2 macrophage) are shown (lower panel). **e** F4/80^+^ cells were depleted by *i.v.* injection of anti-F4/80 mAb or anti-Gr1 mAb on the day − 1, + 5, + 10 and + 15 with respect to tumor cell injection. Along with F4/80^+^ cell depletion, α-GalCer (2 μg/mouse/injection) was given on the day + 1, + 5, + 10, + 15 and + 20. Tumor growth was monitored, and the tumor area was calculated and plotted. *n* = 4–5 mice/group. **f** At day 20, IFN-γ expression in the splenic NKT cells were analyzed and plotted. *n* = 3–5 mice/group. The bar represents s.e.m., and each dot represents an individual mouse. (**a**, **b**, **f**). One-way ANOVA (**e**). Student’s *t*-test (**a**, **b**, **f**). *p < 0.05; **p < 0.01; ns, not significant

Intratumoral macrophages are reported to have M2 phenotype [41]. Since α-GalCer administration increased the IFN-γ secretion and NKT cell frequency in the tumor, we investigated if the increased IFN-γ could lead to polarization of monocyte/macrophages into classically activated or M1-polarized macrophages that may have an anti-tumor function. Our results showed that α-GalCer treatment did not change the frequency of total F4/80^+^CD11b^+^ macrophages in the spleen and tumor (Fig. 5a). Further, based on iNOS staining (a marker for M1 Macrophage) and CD206 staining (a marker for M2 macrophages), we characterized the M1 and M2 macrophages in the tumor and spleen (Additional file 1: Figure S4). Our results showed that frequency of iNOS^+^F4/80^+^CD11b^+^ macrophages (M1 macrophage) significantly increased with α-GalCer treatment while CD206^+^ F4/80^+^CD11b^+^ macrophages (M2 macrophages) was reduced in the spleen as compared to control group (Fig. 5b). This was in line with immunofluorescence data which revealed an increased number of iNOS^+^ M1 macrophage with α-GalCer treatment in the spleen and tumor as compared to control mice (Fig. 5c). Furthermore, α-GalCer treatment also reduced the percentage of CD206^+^ M2 macrophages in the spleen and tumor (Fig. 5d). To investigate if the increased frequency of M1 macrophages in the spleen and tumor was responsible for α-GalCer-induced reduction of tumor growth (Fig. 3a), we depleted the macrophages using anti-F4/80 mAb and monitored the tumor growth. Our results showed that depletion of F4/80^+^ macrophages using anti-F4/80 mAb prevented the α-GalCer-induce inhibition of tumor growth (Fig. 5e). Furthermore, depletion of monocytes using anti-GR1 mAb was less effective as compared to the anti-F4/80 mAb suggesting that differentiated macrophages had a more pronounced effect than Gr1^+^ monocytes to control of tumor growth (Fig. 5e). Depletion of F4/80^+^ cells, Gr-1^+^ cells or treatment with isotype control antibody did not significantly alter the tumor growth kinetics as compared to the control group (Additional file 1: Figure S5). Furthermore, depletion of F4/80 macrophages did not change the α-GalCer-induced IFN-γ production in NKT cells (Fig. 5f). Together, these results suggest that α-GalCer increases the frequency of M1 macrophages in the spleen and tumor, leading to inhibition of tumor growth that is mediated through NKT cells.

## Discussion

NKT cell is a group of unique lymphocytes that are capable of recognizing lipid antigens presented by CD1d molecule. Activation of NKT cells through CD1d induces the release of a wide array of cytokines such as IFN-γ, IL-4, IL-6, IL-10, IL-17, TNF-α, and chemokines such as RANTES, MIP-1α, and MIP-1β [28, 42–44]. Secretion of these molecules by NKT cells contributes an important role in several diseases such as autoimmunity, infection, and tumor immunity. NKT cells have been observed to infiltrate in a different type of human tumors such as myeloma, prostate cancer, colon carcinoma, head and neck cancer, breast tumor, renal cell cancer and melanoma [12, 45–47]. However, few studies have observed an increased infiltration of Vα24 NKT cells in hepatocellular carcinoma and lung cancer [48, 49]. In our study, we found a high infiltration of NKT cells in B16F10 induced melanoma tumor as early as day 5 of tumor growth. However, the frequency of NKT cells in the tumor decreases as cancer progresses to day 13 suggesting that tumor-induced immune suppression might play a crucial role. Yang et al. have suggested that splenic NKT cells are mostly CD62L^high^CD69L^low^ and have memory phenotype [50]. We reported that intratumoral NKT cells expressing lower CD69 and higher CD62L as compared to splenic NKT cells suggesting that tumor-associated NKT cells display memory phenotype. In light of these studies, we also observed that intratumoral NKT cells had reduced proliferation as compared to the splenic NKT cells.

NKT cells can recognize and kill CD1d-expressing tumors such as lymphoma, early myeloma, prostate cancer, medulloblastoma and myeloid leukemia [4]. NKT cells can also exert anti-tumor function by secretion of cytokines that can trans-activate NK cells or modulate the immunosuppressive cells in the tumor such as tumor-associated macrophages [51, 52]. NKT cell-derived IFN-γ has been reported to promote antigen-specific CD8^+^ T cell response in melanoma patients [23, 53]. Prostate cancer and myeloma patients show a significantly reduced frequency of IFN-γ-producing peripheral blood and tumor-infiltrating NKT cells as compared to healthy individuals [12, 45]. Our observation that tumor-infiltrating NKT cells showed lower expression of IFN-γ, TNF-α, and GM-CSF as compared to splenic counterpart suggests that tumor microenvironment may induce changes in NKT cells. Since α-GalCer is known to activate the only type I NKT cells but not type II NKT cells [7], our results with α-GalCer probably responding through directly modulating the type I NKT cells. However, this needs to be further evaluated.

Activation of NKT cells by α-GalCer is reported to inhibit the metastasis in B16F10-induced melanoma, colon carcinoma, and spontaneous sarcomas in p53^−/−^ mice [16, 54, 55]. Our data also show that α-GalCer treatment could also control the tumor growth in an NK1.1^+^ cell-dependent manner. In the lung and liver metastasis model, the anti-metastatic activity of α-GalCer is dependent on IFN-γ-production by NKT cells [19]. In our model, we observed that IFN-γ production by NKT cells was up-regulated upon α-GalCer administration while other cytokine levels were unaltered suggesting that increased frequency and IFN-γ production by NKT cells contribute to inhibiting the tumor growth. A study by Shimizu et al. showed that vaccination of mice with B16F10 tumor cells loaded with α-GalCer (B16/Gal) could protect mice from subsequent tumor challenge. Mechanistically, cross-presentation of α-GalCer-loaded tumor cells by DC could prime CD4^+^ and CD8^+^ T cell response and provide long-lived immunity [56]. Our results also showed that α-GalCer treatment enhance the IFN-γ production in CD8^+^ T cells which might contribute in controlling the tumor growth. However, whether α-GalCer-mediated activation of NKT cells regulates CD4^+^ T cell response is not known. Our results suggest that α-GalCer treatment could increase the IFN-γ^+^CD4^+^ Th1 cells as well as RORγt^+^CD4^+^ Th17 cells (data not shown) and was NK1.1^+^ cell dependent. The increased frequency of IFN-γ-expressing CD4^+^ T cells (Th1 cells) can enhance the anti-tumor function. It has been reported that α-GalCer can lead to an anergy-like state in the NKT cells [57–59]. In contrast, our data showed that repeated low dose of α-GalCer injection promoted the frequency of effector Th1, CD8 T cells, and M1 macrophages and controlled the tumor growth. The difference observed in our results and other studies is may be due to the doses and kinetics of α-GalCer injection and difference in solid tumor model versus the metastatic model of B16F10 melanoma.

Interaction among the NK, NKT cells, and macrophages can shape the immune response [60]. A study by Francesca Bellora et al. showed that activated NK cells could lyse M0 and M2 macrophage while M1 macrophages are resistant to lysis [22]. IL-15 has been shown to protect NKT cells from inhibition by TAM and enhance anti-metastatic activity [52]. However, how NKT cell might alter the frequency macrophage polarization in the tumor microenvironment is not clearly understood. Our data suggest that NKT cell activation by α-GalCer might promote the percentage of iNOS^+^ M1 macrophages while reducing the frequency of CD206^+^ M2 macrophages in the spleen and tumor microenvironment. A study by Song et al. showed that Vα24-NKT cells mediate the anti-tumor function by killing the tumor-associated macrophages [51], and loss of NKT cells promote pancreatic cancer in LSL^KrasG12D/+^ mice through increase M2 macrophages phenotype [61]. Furthermore, depletion of macrophage using anti-F4/80 antibody reverses the beneficial effect of α-GalCer suggesting that α-GalCer-induced higher M1 macrophage frequency in the spleen and tumor play an important role in anti-tumor immunity. Further investigation is warranted to understand the molecular mechanism of NKT cell-mediated differentiation of M1 or M2 macrophages.

In conclusion, our data suggest that α-GalCer activates NKT cells leading to reduction of melanoma tumor by increasing the frequency of M1 macrophage and effector Th1 cells. The findings underscore the potential of α-GalCer as an effective anti-cancer compound.

## Additional file

Additional file 1: **Figure S1.** Anti-NK1.1 antibody (clone PK136) depletes NK cells in the spleen. **Figure S2.** α-GalCer treatment controls tumor growth in an NK cell-dependent manner. **Figure S3.** NK cell depletion abolishes the -GalCer-induce inhibition of tumor growth. **Figure S4.** Gating strategy for M1- and M2-macrophage analysis. **Figure S5.** Depletion of Gr1^+^ and F4/80^+^ cells do not significantly alter the tumor growth. (PDF 2319 kb)

## Data Availability

The dataset used and/or analyzed during the current study are available from corresponding author on reasonable request.
