# Supplementary material for: Natural killer T cell activation increases iNOS+CD206- M1 macrophage and controls the growth of solid tumor
Source: J Immunother Cancer. 2019 Aug 6;7:208. doi: 10.1186/s40425-019-0697-7 (PMC6685184; doi:10.1186/s40425-019-0697-7)
Supplement: Supplementary file 1 — Figure S1. Anti-NK1.1 antibody (clone PK136) depletes NK cells in the spleen. Figure S2. α-GalCer treatment controls tumor growth in an NK cell-dependent manner. Figure S3. NK cell depletion abolishes the -GalCer-induce inhibition of tumor growth. Figure S4. Gating strategy for M1- and M2-macrophage analysis. Figure S5. Depletion of Gr1+ and F4/80+ cells do not significantly alter the tumor growth. (PDF 2319 kb) [file 40425_2019_697_MOESM1_ESM.pdf]

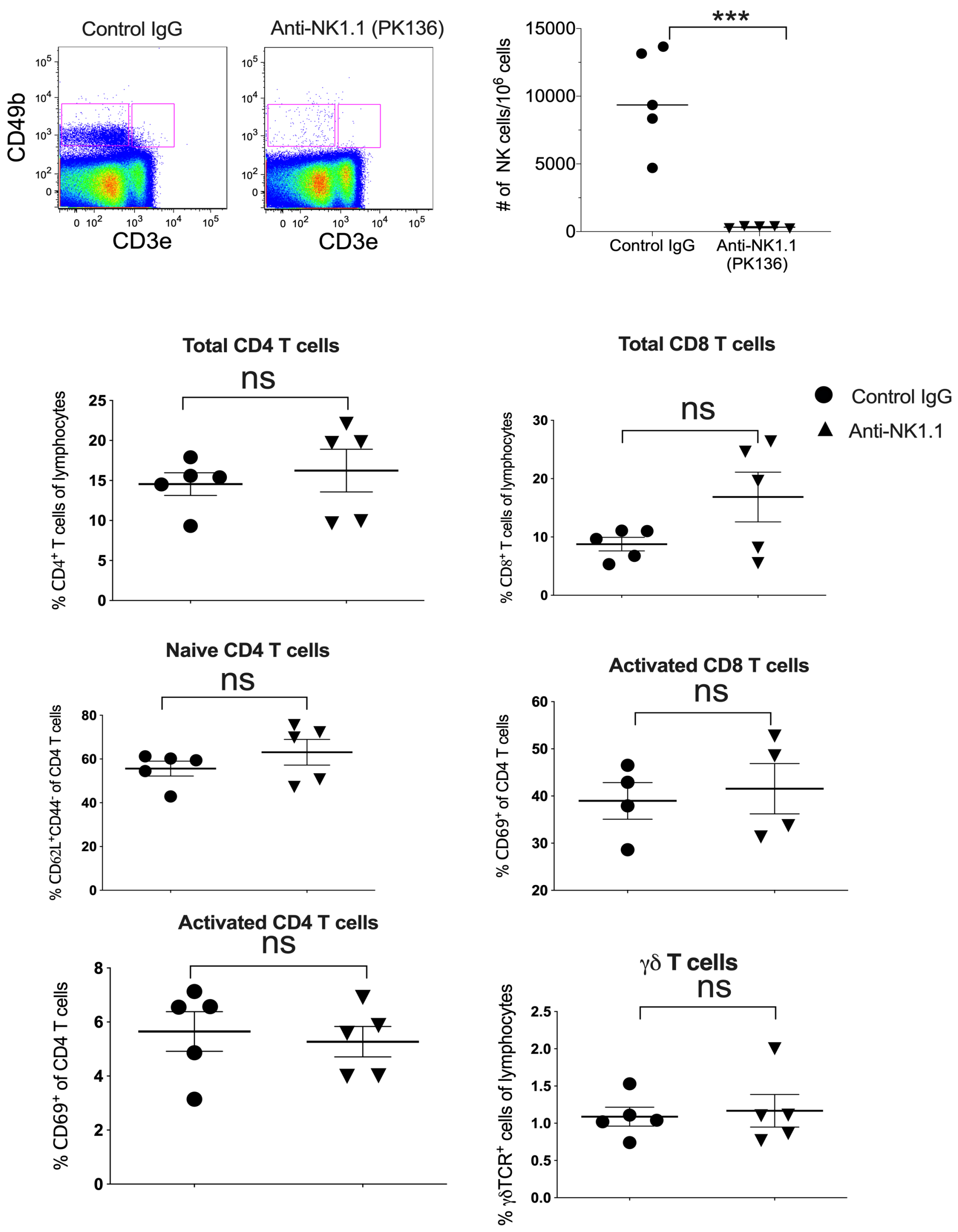

Figure S1: Anti-NK1.1 antibody (clone PK136) depletes NK cells in the spleen. Naïve C57BL6 mice were given s.c. injection of B16F10 cells (1 X 10^6 cells/mouse) and either Isotype control IgG or anti-NK1.1 mAb by *i.v.* injection (100μg/mouse/injection) on day -3, +1, +5, +10, +15 and +20 with respect to tumor cell injection. Animals were sacrificed on day 23, the spleen was harvested and various immune cells were analyzed using flow cytometry. Cells are gated on lymphocyte gate followed by singlet population. Each symbol represents data from individual mice. Student's *t*-test, ns= not significant, ***p<0.0001.

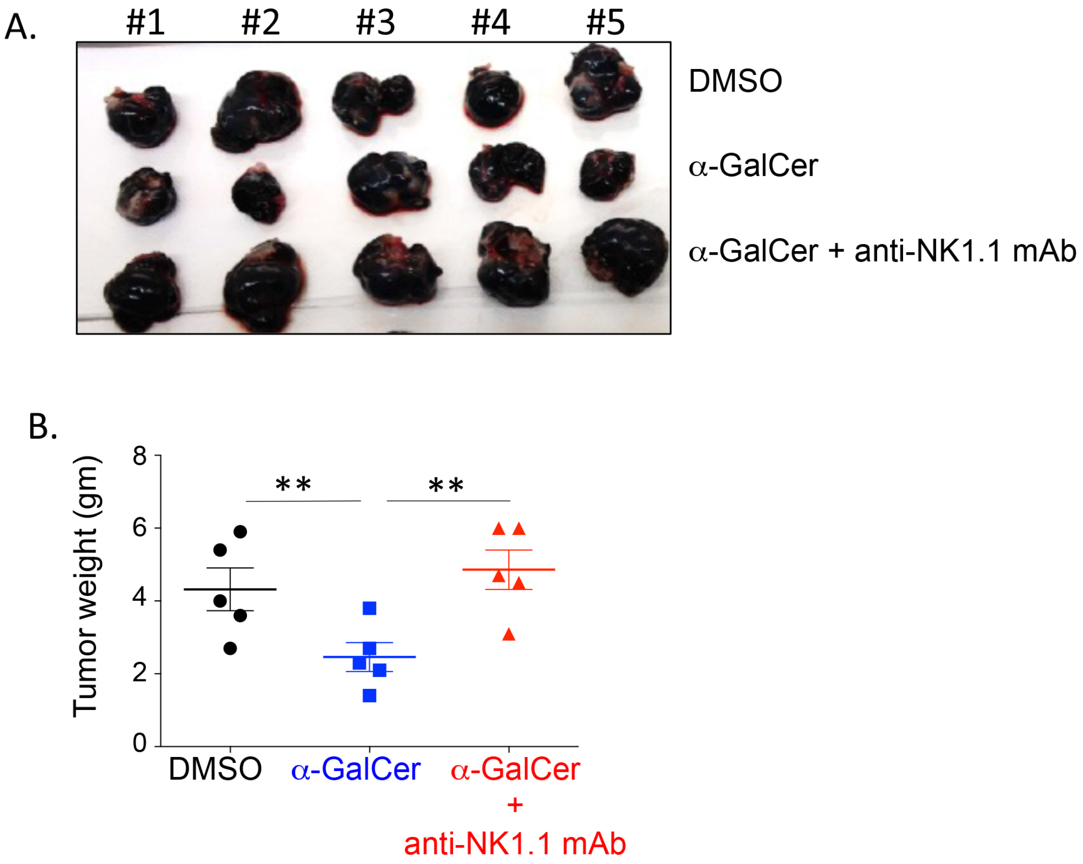

Figure S2: α-GalCer treatment controls tumor growth in an NK cell-dependent manner. Naïve C57BL6 mice were given s.c. injection of B16F10 cells (1 X 10^6 cells/mouse), and NK cells were depleted by *i.v.* injection of NK1.1 mAb (PK136; 100µg/mouse/injection) on day -3, +1, +5, +10, +15 and +20 with respect to tumor cell injection. α-GalCer (2µg/mouse/i.p. injection) was given on day +1, +5, +10, +15 and +20 with respect to tumor cell injection. **(A)** The tumor was excised. The image of each tumor is shown. **(B)** The weight of the tumors was measured and plotted. Each symbol represents the weight tumor from an individual mouse. n=5 mice/group. Student's *t*-test, **p < 0.01.

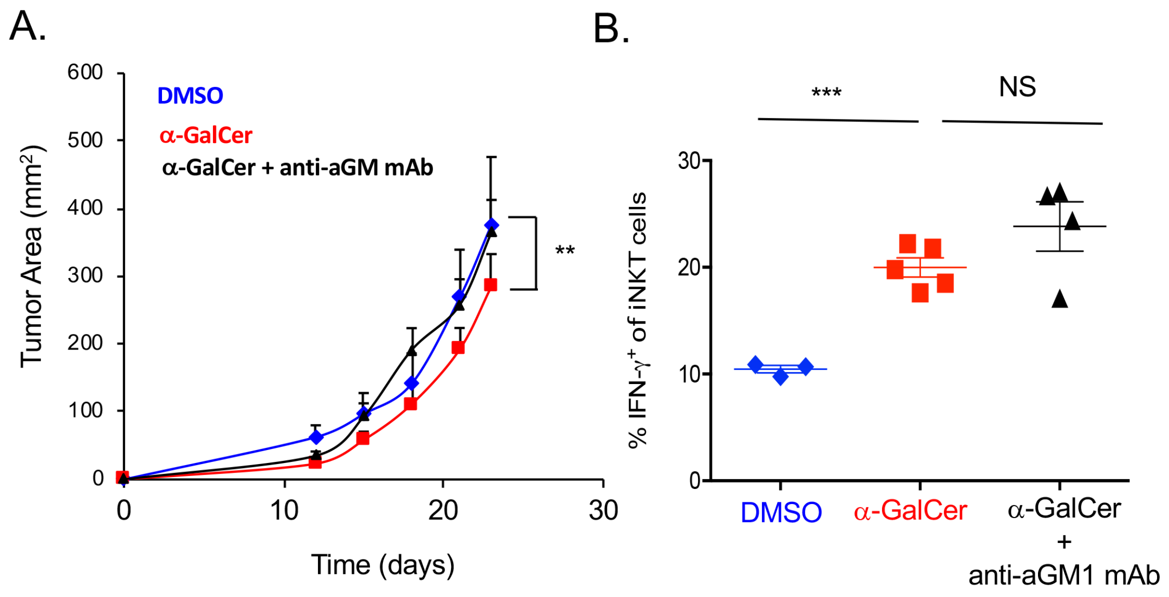

Figure S3: NK cell depletion abolishes the α-GalCer-induce inhibition of tumor growth. Naïve C57BL6 mice were given s.c. injection of B16F10 cells (1 X 10^6 cells/mouse) and NK cells were depleted by *i.v.* injection of polyclonal anti-asialo GM1 antibody (100μg/mouse/injection) on day -3, +1, +5, +10, +15 and +20 with respect to tumor cell injection. α-GalCer (2μg/mouse/i.p injection) was given on day +1, +5, +10, +15 and +20 with respect to tumor cell injection. **(A)** The tumor area was measured and plotted. **(B)** IFN-γ-producing NKT cells in the spleen was analyzed and plotted. Each symbol represents data from an individual mouse. n = 3-5 mice/group. Student's *t*-test, **p < 0.01; ***p < 0.001; NS, not significant.

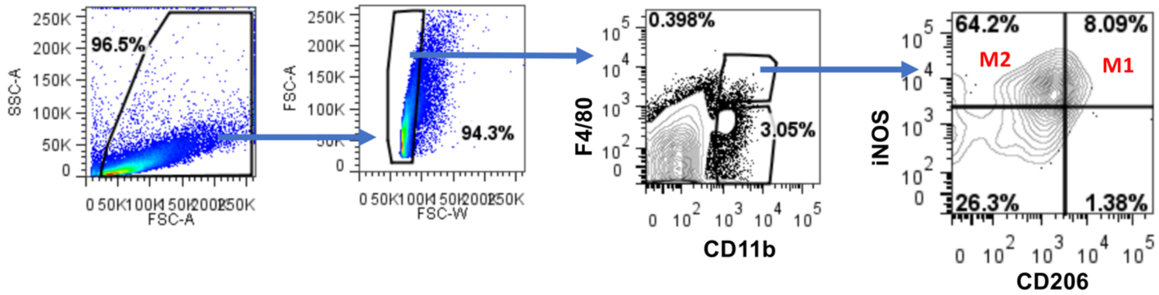

Figure S4: Gating strategy for M1- and M2-macrophage analysis.

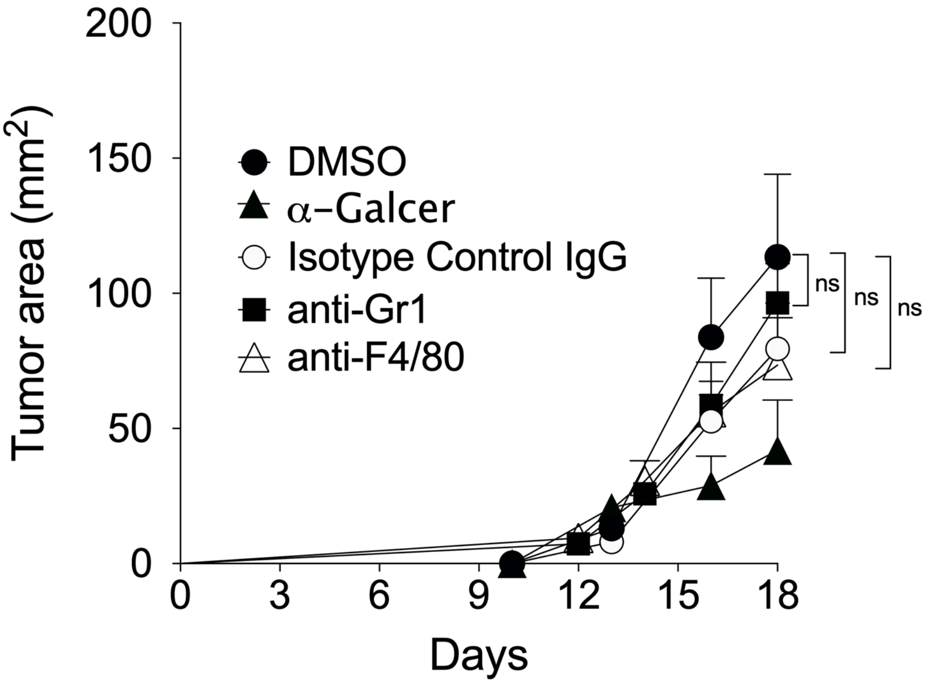

Figure S5: Depletion of Gr1$^+$ and F4/80$^+$ cells do not significantly alter the tumor growth. Naïve C57BL6 mice were given s.c. injection of B16F10 cells (1 X 10^6 cells/mouse) and indicated antibodies by *i.v.* injection (100µg/mouse/injection) on day -3, +1, +5, +10 and +15 with respect to tumor cell injection. α-GalCer (2µg/mouse/i.p injection) was given on day +1, +5, +10, +15 and +20 with respect to tumor cell injection. The tumor area was measured and plotted. n = 6 mice/group. ns= not significant, analyzed by one-way analysis of variance (ANOVA).
